# One step further toward a crop CO2-concentrating mechanism

**DOI:** 10.1093/jxb/erad200

**Published:** 2023-06-27

**Authors:** Justin Findinier, Arthur R Grossman

**Affiliations:** The Carnegie Institution for Science, Biosphere Sciences & Engineering, Stanford, CA 94305, USA; The Carnegie Institution for Science, Biosphere Sciences & Engineering, Stanford, CA 94305, USA; Stanford University, Department of Biology, Stanford, CA 94305, USA

**Keywords:** Bicarbonate, carbonic anhydrase, CCM, engineering, photosynthesis, transporter

## Abstract

This article comments on:

**Förster B, Rourke LM, Weerasooriya HN, Pabuayon ICM, Rolland V, Au EK, Bala S, Bajsa-Hirschel J, Kaines S, Kasili RW, LaPlace LM, Machingura MC, Massey B, Rosati VC, Stuart-Williams H, Badger MR, Price GD, Moroney JV**. 2023. The *Chlamydomonas reinhardtii* chloroplast envelope protein LCIA transports bicarbonate *in planta*. Journal of Experimental Botany **74**, 3651–3666

**The CO**_**2**_ **-concentrating mechanism (CCM) used by eukaryotic algae represents an inorganic carbon pump [Ci: bicarbonate (HCO**_**3**_^**–**^ **), carbon dioxide (CO**_**2**_ **), and carbonic acid (CO**_**3**_^**2–**^ **)] that generates an elevated concentration of CO**_**2**_ **around Rubisco, which promotes carbon fixation. This mechanism, which evolved independently several times, has the potential to be transferred (at least some key activities) into crop species, which could boost agricultural yields and contribute to sustaining a growing world population. One component of the CCM of the unicellular green alga *Chlamydomonas reinhardtii* is the putative chloroplast envelope bicarbonate channel, LCIA. In their study, Förster *et al*. (2023) have exploited heterologous systems defective for concentrating Ci to provide strong evidence that LCIA functions as a channel that facilitates bicarbonate movement into the plastid stroma.**

Photosynthesis is the process by which plants, algae, and some bacteria use sunlight to fix atmospheric CO_2_ and fuel cell growth and metabolism. CO_2_ fixation is performed by Rubisco, an enzyme with a low affinity for CO_2_ that also catalyzes the photorespiratory oxygenation of ribulose-1,5-bisphosphate. Oxygenation results in the production of the potentially toxic metabolite 2-phosphoglycolate; this reaction, which can account for 25% of Rubisco activity in C_3_ plants, becomes more prominent as the cells become depleted of Ci and at elevated temperatures (Hanson and Peterson, 1986). In aquatic environments, the slow diffusion of CO_2_ from the atmosphere into the water body and across cellular membranes may also favor the Rubisco oxygenase reaction. Photorespiratory metabolism recycles 2-phosphoglycolate into 3-phosphoglycerate, which can re-supply the Calvin–Benson–Bassham cycle with carbon backbones; this energetically wasteful pathway uses multiple enzymatic steps involving two or three distinct organelles. As a potential strategy for increasing the yields of crop species, various groups have engineered photorespiratory bypasses that generate increased photosynthetic efficiency and biomass (Cavanagh *et al*., 2022; Nayak *et al*., 2022). Researchers have also focused on improving the specificity of Rubisco for CO_2_ relative to O_2_, although this approach generally results in a slower rate of substrate turnover by the enzyme (Erb and Zarzycki, 2018).

Green algae and cyanobacteria have evolved various types of CCMs that elevate the level of CO_2_ in the vicinity of Rubisco, increasing the carboxylation efficiency. The core CCM activities are bicarbonate transport and rapid bicarbonate–CO_2_ interconversion catalyzed by carbonic anhydrases (CAs). Once transporters bring bicarbonate physically close to Rubisco, this anion is converted to CO_2_ by CA, providing Rubisco with high levels of substrate for fixation. The uptake and interconversion of Ci species when Ci becomes limiting to the cells are often accompanied by changes in intracellular architecture, which probably further improve the efficiency of the CCM.

While some plants have a C_4_-type, biochemical CCM, C_3_ crop plants are not able to concentrate Ci. Therefore, instead of only trying to improve plant productivity through the modification of endogenous enzymes/processes, researchers are attempting to engineer plants for efficient acquisition of Ci and sustaining an elevated CO_2_/O_2_ ratio in the vicinity of Rubisco, which would favor carboxylation over oxygenation. To achieve this goal, genes encoding core CCM functions are being introduced from cyanobacteria and microalgae into vascular plants.

When cyanobacteria experience a low CO_2_ environment, they assemble a new suborganelle delimited by a proteinaceous shell. This suborganelle, the carboxysome, houses tightly packed Rubisco, specific linker proteins, and a CA. The proteinaceous shell of the carboxysome limits CO_2_ leakage and diffusion from the site of fixation (reviewed in Liu, 2022). The synthesis of the carboxysome parallels the induction of plasma membrane transporters that actively pump bicarbonate from the environment into the cytoplasm of the cell. In green algae such as *Chlamydomonas reinhardtii* (Chlamydomonas), the CCM is often dependent on the development of a pyrenoid in the chloroplast (reviewed in Adler *et al*., 2022). The Chlamydomonas pyrenoid, a suborganelle functionally analogous to carboxysomes, is packed with Rubisco, surrounded by a starch shell, and traversed by thylakoid membranes. Under low CO_2_ conditions, the lumenal α carbonic anhydrase CAH3 concentrates in the thylakoid membranes within the pyrenoid matrix where it supplies Rubisco with CO_2_. The starch sheath that surrounds the pyrenoid may be comparable with the carboxysome’s shell, limiting leakage of CAH3-generated CO_2_. Overall, pyrenoid development and other low CO_2_-associated processes such as peripheral mitochondria repositioning, induction of genes encoding putative bicarbonate transporters that are targeted to the plasma membrane (HLA3; Yamano *et al*., 2015), the chloroplast inner envelope membrane (LCIA; Mariscal *et al*, 2006; Yamano *et al*., 2015), and thylakoid membranes (Mukherjee *et al*., 2019), and the routing of bicarbonate to the pyrenoid-localized thylakoids where it can be converted to CO_2_ by CAH3 (Fig. 1) are all necessary activities associated with an energized biophysical CCM.

**Fig. 1.**
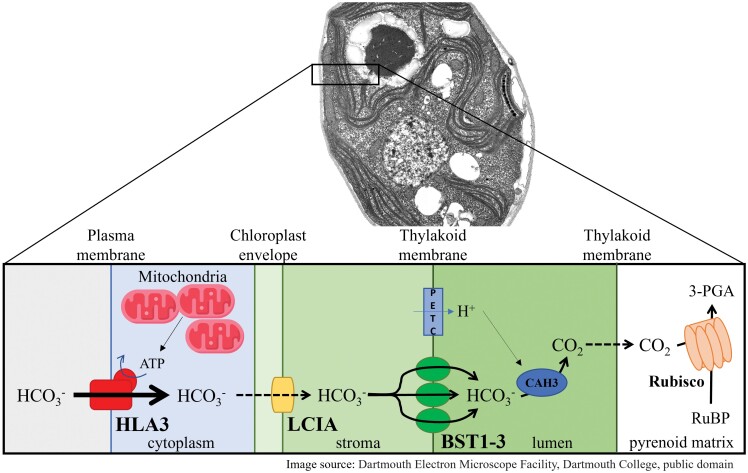
The intricate functioning of the bicarbonate (HCO_3_^–^) gradient in Chlamydomonas. To deliver CO_2_ to Chlamydomonas Rubisco, which is concentrated in the pyrenoid, at least three bicarbonate transport systems are used; one on the plasma membrane, another on the chloroplast inner envelope, and a third on the thylakoid membranes (Adler *et al*., 2022). These transporters can generate a Ci gradient extending from the environment to the pyrenoid where the CO_2_ is fixed. The plasma membrane transporter HLA3 is an ABC transporter that actively pumps bicarbonate into the cytosol of the cell. The ATP necessary for energization of bicarbonate import across the plasma membrane is thought to be derived from fixed carbon synthesized in the chloroplast that moves to the mitochondria where it is respired (Burlacot *et al*., 2022); the tight apposition of the mitochondria with both the plasma membrane and chloroplast envelope that occurs in cells experiencing low Ci would allow for efficient exchange (reduced distances) of metabolites between different intracellular compartments. The chloroplast envelope transporter LCIA, characterized in the study discussed here, is a bicarbonate channel that facilitates diffusion of bicarbonate into the chloroplast which is driven by the concentration gradient created by active pumping of bicarbonate at the plasma membrane. Furthermore, once in the stroma, bicarbonate enters the lumen of the thylakoid membranes through a family of three bestrophin-like bicarbonate transporters, BST1–BST3 (Mukherjee *et al*., 2019). This import is probably powered by the proton motive force (pmf) across the thylakoid membranes that is generated by photosynthetic electron transport. The pmf (low pH in the lumen) also promotes dehydration of bicarbonate in the lumen, yielding CO_2_ which rapidly diffuses across the thylakoid membrane and is fixed by Rubisco in the pyrenoid by bonding to ribulose-1,5-bisphosphate (RuBP) to produce 3-phosphoglycerate (3-PGA). The pmf, therefore, also participates in maintaining a low stromal bicarbonate concentration, allowing its continuous diffusion through the LCIA channel. Image source: Dartmouth Electron Microscope Facility, Dartmouth College, public domain.

The Chlamydomonas chloroplast envelope transporter LCIA, studied by Förster *et al*. (2023), belongs to the NAR1 family of nitrite/formate transporters. Its expression profile and mutant phenotype strongly indicate a role in low CO_2_ acclimation, especially when bicarbonate is the main Ci form. Expression of *LCIA* in Xenopus oocytes (Mariscal *et al*., 2006; Atkinson *et al*., 2016) has suggested its function in bicarbonate transport, but no clear evidence has been acquired *in planta*. Förster *et al*. explored the function of LCIA using heterologous systems defective for growth at ambient levels of CO_2_. In *Escherichia coli*, CA Can/YadF maintains a pool of bicarbonate in the cytoplasm that is necessary to support essential processes such as anaplerotic metabolism; a knockout mutant of Can/YadF is unable to grow unless supported by high levels of atmospheric CO_2_ (Merlin *et al*., 2003). In *Arabidopsis thaliana* (Arabidopsis), the βCA1 and βCA5 proteins participate in chloroplast carbon metabolism by supplying bicarbonate to the enzyme acetyl-CoA carboxylase, which catalyzes the first step in fatty acid synthesis. βCA5 is mostly expressed in root tissues, and a null mutant for this protein shows severely impaired growth at ambient CO_2_ levels (Weerasooryia *et al*., 2022). Additionally, the *βca5* mutant was recently used to show that the Chlamydomonas protein LCIB has CA activity (Kasili *et al*., 2023). Förster *et al*. (2023) took advantage of the phenotypes of these *E. coli* and Arabidopsis mutants to assay the capacity of LCIA to support the growth of these strains at ambient levels of CO_2_. LCIA, targeted to the plasma membrane of the *E. coli* mutant and the chloroplast envelope of the Arabidopsis mutant, substantially restored their growth under ambient CO_2_ conditions. Using the elegant silicone oil layer centrifugation assay (Badger *et al*., 1980), LCIA was also shown to directly improve bicarbonate uptake in *E. coli*.

After obtaining evidence that LCIA functions *in vivo*, the authors investigated the possibility of a fitness effect of LCIA in wild-type tobacco plants. Conflicting results have been reported regarding the impact of LCIA expression in plants. Nölke *et al.* (2019) observed improved tobacco growth when the plants expressed either LCIA or the lumenal carbonic anhydrase, CAH3 (using a small mAb54 epitope tag). On the other hand, Atkinson *et al*. (2016) did not observe an effect of LCIA–green fluorescent protein (GFP) when it was expressed and localized to the chloroplast envelope in wild-type Arabidopsis. Here, Förster *et al*. assessed several photosynthetic parameters in tobacco plants expressing LCIA–GFP but, unlike Nölke *et al* (2019), did not observe enhanced fitness. This absence of enhancement, if the introduced LCIA–GFP is fully functional in the heterologous system, most probably reflects a lack of coordination with other plasma membrane and thylakoid membrane transporter activities in tobacco (Fig. 1).

Together with bacteria and yeast knockout strains (Kasili *et al*., 2023), Förster *et al*. (2023) now describe new heterologous model organisms that can interchangeably be used to assay the functionality of potential bicarbonate transporters and CAs. The Arabidopsis model is the most well characterized chassis for identifying proteins involved in improving Ci use efficiency in plants. It has been proposed that increasing the stromal Ci concentration can yield up to a 9% increase in plant biomass (McGrath and Long, 2014). This increase could potentially be achieved by introducing an active bicarbonate transporter into the chloroplast envelope and/or a stromal CA, which would trap CO_2_ in the stroma as bicarbonate. The Arabidopsis *βca5* mutant will surely help build a list of candidate bicarbonate transporters and CAs and reveal their catalytic characteristics *in planta*.

Engineering an efficient land plant CCM-like activity will probably require multiple proteins, either from a single host, such as Chlamydomonas or *Synechococcus*, or from multiple systems. For constructing a CCM in crop plants, the energetic requirements to support the system must also be considered, including active Ci pumping (Fig. 1) (Burlacot *et al*., 2022). A recent model suggests that introducing the stromal components of the Chlamydomonas CCM (pyrenoid components+stromal CA) into plants could lead to more efficient Ci utilization (Fei *et al*., 2022). Therefore, introduction of LCIA into plants might not be the best approach for establishing increased Ci use efficiency; the introduction of a highly active stromal CA that couples with CO_2_ fixation and the generation of a Ci gradient might be more effective in promoting bicarbonate diffusion across the envelope-localized LCIA channel. Once an initial improvement in plant Ci utilization is achieved, further tailoring would be required for optimal performance.

While our knowledge of structural components of the algal and cyanobacterial CCM is sufficiently advanced to guide initial efforts in building a pyrenoid or a carboxysome *in planta*, moving toward the establishment of an efficient CCM in crop plants will benefit from an increased understanding of structural and functional aspects of the CCM and the energization of the system.

## Data Availability

No new data were presented in this article.
